# 

**Published:** 1805-03-01

**Authors:** 


### To CORRESPONDENTS.

Communications are received from Dr. Girdlestone, Mr. Whately, Mr. Ring, Mr. Cuming, Mr. Harrison, Mr. Simpson, and W. L.

